# Analysis of neuroanatomical differences in mice with genetically modified serotonin transporters assessed by structural magnetic resonance imaging

**DOI:** 10.1186/s13229-018-0210-z

**Published:** 2018-04-10

**Authors:** Jacob Ellegood, Yohan Yee, Travis M. Kerr, Christopher L. Muller, Randy D. Blakely, R. Mark Henkelman, Jeremy Veenstra-VanderWeele, Jason P. Lerch

**Affiliations:** 1 Mouse Imaging Centre (MICe), Hospital for Sick Children, 25 Orde Street, Toronto, Ontario M5T 3H7 Canada; 2 Department of Pharmacology, Vanderbilt University, Nashville, TN 37235 USA; 3 Department of Psychiatry, Vanderbilt University, Nashville, TN 37235 USA; 4 Department of Medical Biophysics, University of Toronto, Toronto, ON M5S Canada; 5 Department of Biomedical Science and Brain Institute, Florida Atlantic University, Jupiter, FL 33431 USA; 6 Department of Psychiatry, Columbia University, New York, NY 10027 USA

**Keywords:** Serotonin, Slc6a4, 5-HT, 5HTT, Magnetic resonance imaging, Neurodevelopment, Brain, Dorsal raphe

## Abstract

**Background:**

The serotonin (5-HT) system has long been implicated in autism spectrum disorder (ASD) as indicated by elevated whole blood and platelet 5-HT, altered platelet and brain receptor and transporter binding, and genetic linkage and association findings. Based upon work in genetically modified mice, 5-HT is known to influence several aspects of brain development, but systematic neuroimaging studies have not previously been reported. In particular, the 5-HT transporter (serotonin transporter, SERT; 5-HTT) gene, *Slc6a4*, has been extensively studied.

**Methods:**

Using a 7-T MRI and deformation-based morphometry, we assessed neuroanatomical differences in an *Slc6a4* knockout mouse on a C57BL/6 genetic background, along with an *Slc6a4* Ala56 knockin mouse on two different genetic backgrounds (129S and C57BL/6).

**Results:**

Individually (same sex, same background, same genotype), the only differences found were in the female *Slc6a4* knockout mouse; all the others had no significant differences. However, an analysis of variance across the whole study sample revealed a significant effect of *Slc6a4* on the amygdala, thalamus, dorsal raphe nucleus, and lateral and frontal cortices.

**Conclusions:**

This work shows that an increase or decrease in SERT function has a significant effect on the neuroanatomy in 5-HT relevant regions, particularly the raphe nuclei. Notably, the *Slc6a4* Ala56 knockin alone appears to have an insignificant, but suggestive, effect compared to the KO, which is consistent with *Slc6a4* function. Despite the small number of 5-HT neurons and their localization to the brainstem, it is clear that 5-HT plays an important role in neuroanatomical organization.

**Electronic supplementary material:**

The online version of this article (10.1186/s13229-018-0210-z) contains supplementary material, which is available to authorized users.

## Background

The brain serotonergic system influences multiple processes during development and throughout life, including neurogenesis, programmed cell death, cell migration, dendritic and axonal development, synaptogenesis, and synaptic plasticity [1–4]. Serotonergic neurons are among the earliest in the brain to be specified for neurotransmitter phenotype and project from the raphe nuclei, which flank the midline along the rostral-caudal extension of the midbrain and brainstem [1–4]. Based on the distribution of the main projections from the raphe, two clusters of raphe nuclei can be defined, a rostral and a caudal group [1, 5–8]. The rostral group projects mainly to the forebrain and is composed of the caudal linear, the dorsal raphe, and median raphe nuclei. This rostral group accounts for 85% of all the serotonergic neurons in the brain [1]. The caudal group primarily projects to the brainstem, cerebellum, and spinal cord [1]. Relative to the rest of the cell bodies in the brain, which number in the billions, serotonin (5-HT) neurons account for approximately 1/1,000,000 of all neurons in the brain [9]. While this is a small portion of the overall brain neurons, the serotonergic neurons project throughout the brain, touching multiple systems.

The 5-HT system has been linked to several disorders, including, but not limited to, obsessive compulsive disorder (OCD), anxiety, depression, schizophrenia, Down syndrome, and autism spectrum disorder (ASD) [10]. In ASD, the disruption of the serotonergic system is one of the more consistent and well-replicated findings [11–13]. In fact, elevated blood 5-HT levels in a group of 50 children with autism was first reported in 1961 [14], and this hyperserotonemia in ~ 25% of autistic patients has been consistently reported [11, 15]. Of the 800+ genes that have been associated with autism [16, 17], there are several related to 5-HT signaling, including the antidepressant-sensitive 5-HT transporter (serotonin transporter, SERT; 5-HTT) gene (*SLC6A4*) [18] and the integrin β3 gene (*ITGβ3*) [19], a SERT-interacting protein that influences 5-HT levels in the periphery and in the brain [20–22]. SERT is the primary mechanism of 5-HT inactivation, responsible for reuptake of the neurotransmitter into the presynaptic 5-HT neuron, where it can be repackaged or metabolized [23]. Therefore, changes in SERT expression and function alter extracellular 5-HT levels and signaling at 5-HT receptors.

The SERT gene has been extensively studied in the human population, and over 20 different gene variants have been found [24], the most studied of which is the functional promoter length polymorphic repeat (5-HTTLPR) [18], which has also been linked recently in a study of amygdala cortex connections [25]. An additional functional variant includes a variable number of tandem repeats (VNTR) in intron 2 (STin2) [26]. Overall results of those gene variants have been inconclusive; however, there appears to be mounting evidence for a linkage in the 17q11.2 region, which includes the SERT gene [27]. Regardless, it had been speculated that optimal *Slc6a4* activity may need to be highly regulated [28], i.e., both high and low *Slc6a4* activity could correlate with illness susceptibility. We would hypothesize, however, that changes in *Slc6a4* function may lead to disparate or even opposing morphological changes throughout the brain. For instance, the loss of SERT causes disruptions in the organization of the barrel fields in the somatosensory cortex of the mouse [29].

In 1998, the first *Slc6a4* knockout (KO) mouse was created [30], and in that study the authors confirmed that 5-HT levels were 60–80% decreased in the brainstem, frontal cortex, hippocampus, and striatum, likely due to diminished synthesis and recycling of 5-HT. Further, these mice demonstrate a 50% reduction in serotonergic cell number in the dorsal raphe nucleus [31]. *Slc6a4* KO mice display increased anxiety-like behaviors, reduced aggression, and exaggerated stress responses [32]. Despite adult brain expression being limited to 5-HT neurons, SERT is transiently expressed in a number of sensory regions as well as in the prefrontal cortex. Accordingly, disruption of SERT function during development has been linked to changes in the architecture and function of the somatosensory cortex and the medial prefrontal cortex [29, 33–35].

Linkage findings in autism spectrum disorder pointed to the chromosome 17q11 region that contains the SERT gene, and multiple amino acid variants in the SERT gene, including Gly56Ala, which increase 5-HT uptake, have been identified in the families with the strongest evidence of linkage [27]. A *Slc6a4* Ala56 knockin (KI) mouse was created and initially found hyperserotonemia, altered CNS 5-HT system function, and changes in social and repetitive behaviors [36] To examine the effects of genetic background, these animals were backcrossed from the initial 129S6 inbred strain to the C57BL/6 inbred strain frequently used for behavioral experiments [37]. Multiple phenotypes were sensitive to different backgrounds. Additionally, neuroanatomy has also been found to be sensitive across inbred strains [38].

With the long-standing association between 5-HT and autism and the well-described effects of 5-HT on neurodevelopment, it is perhaps surprising that the direct relationship between 5-HT and mesoscopic morphological changes throughout the brain has not been systematically investigated. Volume differences in several mouse models related to both autism and the 5-HT system have been examined previously, including an *Itgb3* KO, which encodes a SERT-binding partner [39]. Also, a recent investigation of 26 mouse models related to autism revealed that the neuroanatomical differences clustered into three distinct groups with similar neuroanatomical phenotypes [40]. While that study includes the male animals from the *Slc6a4* KO and *Slc6a4* Ala56 KI models, that paper did not examine the individual models in detail but only the similarities and differences across multiple autism-related mouse models. Given the effect these genetic manipulations on 5-HT levels in the two different models, we hypothesize that changes in brain structure may occur as a direct result of differing levels of serotonin fiber projections brought about by the manipulation. Therefore, the purpose of the current study is to examine, in detail, the specific neuroanatomical differences caused by the different *Slc6a4* mutations.

## Methods

### Mice

Three different mouse lines were used in this study: (1) homozygous *Slc6a4* knockout mice (*Slc6a4* KO) and control animals (C57Bl6/J) were purchased directly from The Jackson Laboratory (JAX #008355, B6.129(Cg)-*Slc6a4*^tm1Kpl^/J and JAX #000664, C57Bl6/J), (2) and (3) *Slc6a4* Ala56 knockin (KI) mice on different inbred strain backgrounds, namely C57BL6/J (B6) and 129S6/SvEvTac (129). *Slc6a4* Ala56 KI mice were created as previously described [28, 36], and their colony was maintained in Vanderbilt University in Nashville, TN, USA. In total 121 mice were examined in this study. Thirty-nine were in the *Slc6a4* KO group, 19 wild-type (WT, 9M and 10 F) and 20 *Slc6a4* KO (10M, 10F); 40 were in the *Slc6a4* Ala56 KI (B6) group, 20 WT (10M and 10F) and 20 *Slc6a4* Ala56 KI (10M and 10F); and 42 were in the *Slc6a4* Ala 56 KI (129) group, 21 WT (11M and 10F) and 21 *Slc6a4* Ala56 KI (11M and 10F). All experimental mice were 60 ± 2 days old.

### Perfusions

Perfusions were either performed on site at the Mouse Imaging Centre (MICe) in Toronto, ON, Canada, for the *Slc6a4* KO mice, or at Vanderbilt University prior to being shipped overnight to MICe. The details of the perfusion protocol have been previously discussed at length [41, 42]. Briefly, mice are anesthetized with a ketamine/xylazine mix or pentobarbital and then intercardially perfused with 30 mL of 0.1 M PBS containing 10 U/mL heparin and 2 mM Prohance (Bracco Diagnostics Inc., a gadolinium contrast agent) followed by 30 mL of 4% paraformaldehyde (PFA) also containing 2 mM Prohance. After perfusion the mouse was decapitated and the skin, ears, and lower jaw were removed. The brain is left within the skull to minimize any deformations and is first incubated in the 4% PFA/Prohance solution overnight. Then, prior to scanning, the brain was incubated for an additional 7 days, at a minimum, in a solution of PBS, 2 mM ProHance, and 0.02% sodium azide, in order to equalize the contrast agent.

### Magnetic resonance imaging

A 7.0-T MRI scanner (Agilent Inc.) was used to acquire all images. For the anatomical scan, a 40-cm inner bore diameter gradient was used (max gradient 30 G/cm) in conjunction with a custom-built solenoid coil array to image 16 samples in parallel [42, 43].

### Anatomical scan

In order to assess the volume differences throughout the brain, a T2-weighted 3D fast spin echo (FSE) sequence was used and is designed for optimized gray/white matter contrast. The sequence parameters for this scan are as follows: TR = 2000 ms, echo train length = 6, echo spacing = 10 ms, with the center of k-space acquired on the fourth echo, TE_eff_ = 42 ms, field of view (FOV) 14 mm × 28 mm × 25 mm, and a matrix size of 250 × 504 × 450, which yields an isotropic (3D) resolution of 56 μm. In the first phase encode dimension, consecutive lines of k-space were assigned to alternating echoes to move discontinuity-related ghosting artifacts to the edges of the FOV [44]. This sequence, therefore, involves an oversampling of k-space in the phase encode direction by a factor of 2 to avoid the interference of ghosting artifacts in the main image, which yields a FOV of 28 mm that is subsequently cropped to 14 mm after reconstruction. Total imaging time for this sequence was 11.7 h.

### Registration and analysis

Deformation-based morphometry (DBM) was used to analyze the volume differences throughout the brain. DBM was performed by registering the mouse brains together through a series of linear (6 parameter fit followed by a 12 parameter fit) and nonlinear fits. After the registration pipeline, all scans are then resampled with the appropriate transform and averaged to create a population atlas representing the average anatomy of the study sample. All registrations were performed with a combination of mni_autoreg tools [45] and advanced normalization tools (ANTs) [46, 47]. The result of the registration is to have all the scans deformed into alignment with each other in an unbiased fashion. This allows for the analysis of the deformations required to take each individual mouse’s anatomy into the final atlas space. The goal is to model how the deformation fields relate to the genotype [48, 49]. The Jacobian determinants of the deformation fields are then used as measures of volume at each voxel. The quantification of the absolute and relative DBM changes are measured from these Jacobians, including the diffeomorphic alignment plus the affine changes for the absolute volume calculation and the diffeomorphic warp alone for the relative volumes. Regional volume differences are then calculated by warping a pre-existing classified MRI atlas onto the population atlas, which allows the calculation of regional volumes across the brain. The classified MRI atlas includes 159 different structures and incorporates three separate pre-existing atlases: (1) delineates 62 different structures throughout the brain including subcortical white and gray matter structures, corpus callosum, striatum, and thalamus [50]; (2) further divides the cerebellum into its various regions, individual lobules, white and gray matter, and the deep cerebellar nuclei [51]; and (3) divides the cortex into 64 different regions, including areas of the cingulate cortex and primary motor and somatosensory cortices [52]. The total brain volume and seven summary regions are also assessed including the cortex (as a whole), cerebellum, ventricles, brainstem, olfactory bulbs, cerebral white matter, and cerebral gray matter. Multiple comparisons were controlled for using the false discovery rate (FDR) [53].

### Additional Slc6a4 comparisons

As the *Slc6a4* Ala56 KI mice and *Slc6a4* KO mice were acquired from different colonies (Jackson Labs for the *Slc6a4* KO mice and Vanderbilt University for the *Slc6a4* Ala56 KI mice), in order to assess the effect of *Slc6a4* across all the mice in this study, we needed to normalize one of the studies to the other using the WT C57Bl6/J mice, since this would account for colony effects between groups. This was done by calculating the differences in the C57Bl6/J WT from the *Slc6a4* Ala56 KI (B6) group acquired from Vanderbilt and the C57Bl6/J WT from Jackson Labs. After the standardization (beta) coefficient was calculated between the two WT groups during the registration process, the calculated 3D scalar value was applied to the full *Slc6a4* KO group (WT and *Slc6a4* KO mice) used in this study to normalize that group to the *Slc6a4* Ala56 KI groups. That normalization applied to the *Slc6a4* KO mice takes into account colony effects between sites and allows a comparison across all mice in this study to determine the variance caused by the *Slc6a4* gene. Variance in the *Slc6a4* gene was assessed across groups using an ANOVA and co-varying for sex and background (~Background + Sex + Genotype).

### Assessment of the dorsal raphe nucleus (DRN) connectivity

To determine whether the *Slc6a4* neuroanatomical phenotype is consistent or related to specific fiber tracts projecting out of the DRN, we compared the absolute *F*-statistic map with neuronal projection data from the Allen Institute [54, 55] after aligning our dataset to the Allen Institute’s Common Coordinate Framework (CCFv3) reference atlas (http://mouse.brain-map.org/static/atlas).

Projection density data derived from 3D, high resolution, whole-brain two-photon microscopy images of neuronal tracers injected into a variety of brain regions were obtained from the Allen Institute’s publicly available API. Specifically, this data is from mouse brains injected with a recombinant adeno-associated viral anterograde tracer that expresses EGFP. Original resolution data is gridded at 50 μm and summarized as the number of voxels within the 50-μm grid that contained a tracer signal (“projection density”). Since data from the connectivity experiments are obtained by injecting only in the right hemisphere, whereas volume changes are bilateral, bilateral fiber tract connectivity is estimated by reflecting each 3D tracer dataset across the sagittal midline plane and merging the data with its mirrored pair by taking the maximum projection density value at each voxel. Of the connectivity experiment datasets downloaded, five consisted of the DRN as the primary injection structure. These five experiments are further merged together by taking the maximum projection density value at each voxel. To determine the association between volume differences and DRN-related connectivity, the absolute *F*-statistic map, taken from the assessment of the variance in the *Slc6a4* gene, is thresholded at an FDR of *q* < 0.05 (*F* > 4.56) and compared with the merged DRN projection density image that was thresholded at 0.1. All voxelwise analyses were carried out within a mask of the whole brain but excluded voxels in the seed region to avoid selection bias.

## Results

**Fig. 1:**
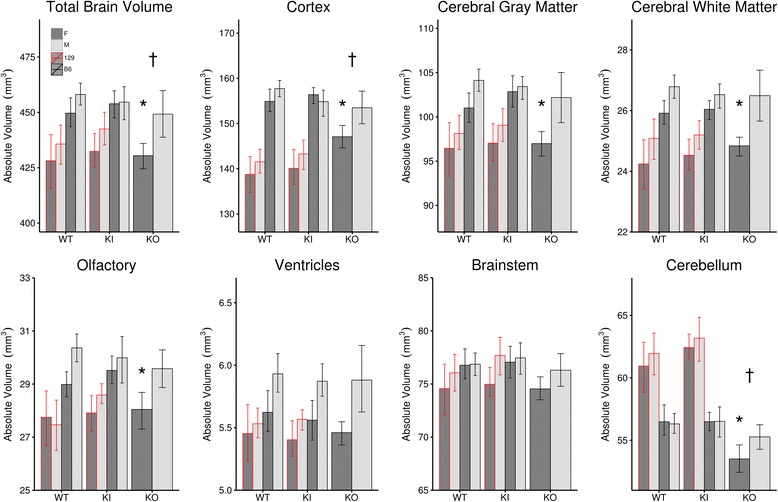
Bar graphs showing the absolute volume for the total brain and seven summary regions encapsulating the entire brain volume. The asterisk represents a significant difference between the single sex group versus its same background wild-type counterpart. The dagger symbol represents a significant difference between the full group (males and females) versus its same background wild-type. *Slc6a4* KO (*N* = 39, 19 WT (9M, 10F), 20 KO (10M, 10F); *Slc6a4* Ala56 KI (B6) (*N* = 40, 20 WT (10M, 10F), 20 KI (10 M, 10F); *Slc6a4* Ala 56 KI (129) (*N* = 42, 21 WT (11M, 10F), 21 KI (11M, 10F)

The first assessment for differences in neuroanatomy was the total brain volume, in which no differences were found in the *Slc6a4* Ala56 KI mice. This was true for the full group, males or females separately, and on both backgrounds (B6 or 129). On the other hand, the *Slc6a4* KOs had a smaller total brain volume for the full group (448 ± 17 mm^3^ for KO versus 462 ± 15 mm^3^ for the WT B6, *q* = 0.04, where q is the FDR-corrected *p* value), largely driven by the female mice (439 ± 10 mm^3^ for KO versus 459 ± 19 mm^3^ for WT, *q* = 0.03), whereas the male-only group had no significant difference in total brain volume (457 ± 18 mm^3^ for KO versus 466 ± 10 mm^3^ for WT, *q* = 0.66) (see Fig. 1). Assessment of the seven summary regions revealed that the total brain volume difference that we were seeing in the full group of *Slc6a4* KO mice was driven largely by the cortex (− 3.8%, *q* = 0.02) and cerebellum (− 3.5%, *q* = 0.01). While the whole brain volume of the female mice was found to be significantly smaller, the female *Slc6a4* KO mice had additional differences in the olfactory (− 4.3%, *q* < 0.05) cerebral gray (− 4.1%, *q* < 0.05) and white matter (− 4.5%, *q* = 0.04) regions. Figure 1 highlights the differences in these summary regions across groups, which includes the normalized *Slc6a4* KO group for comparison. When the relative volume of the summary regions is examined (i.e., each region is measured as a percentage of total brain volume), no significant differences were found. Therefore, the differences we observed in the female *Slc6a4* KO mice indicate that the brains are *almost* uniformly smaller.

**Fig. 2:**
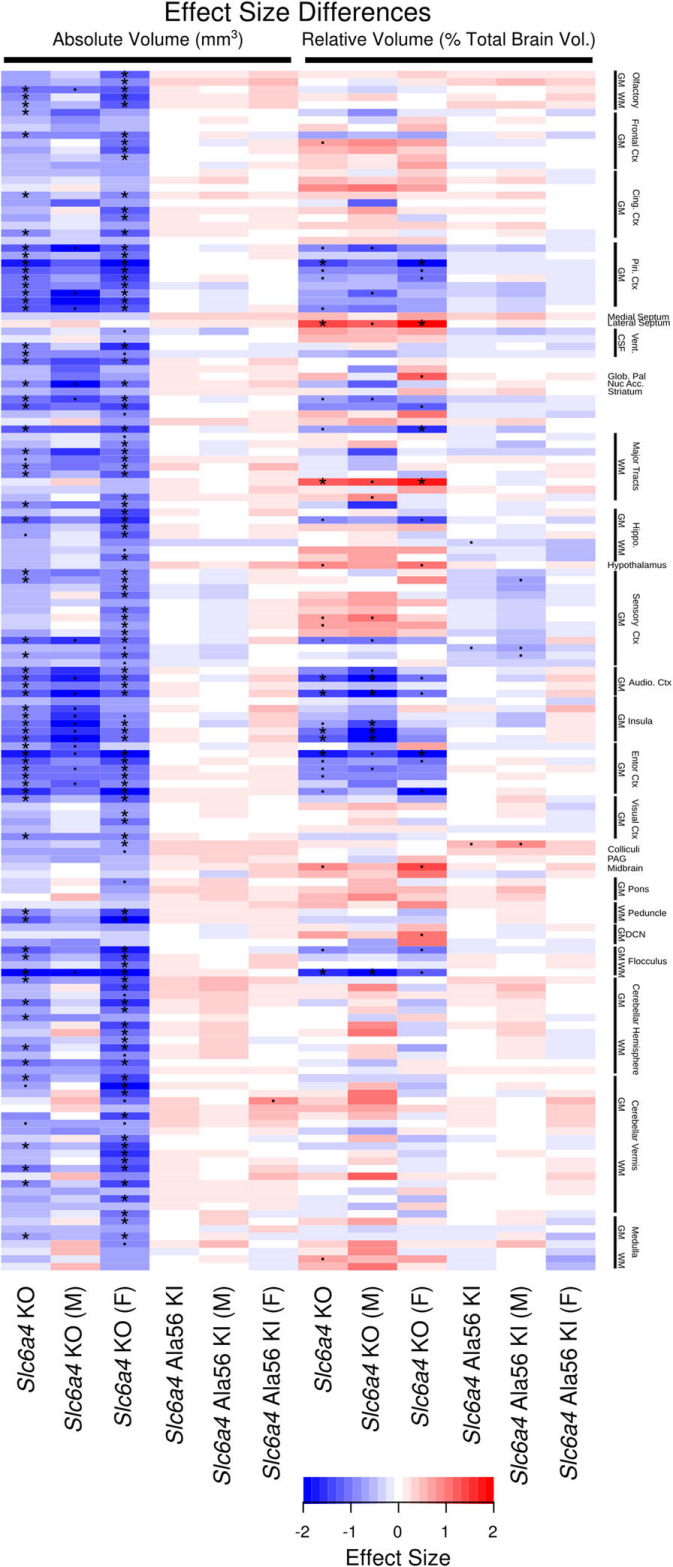
Heatmap showing the effect size differences in 159 different regions throughout the brain for the Slc6a4 KO group and the full Slc6a4 Ala56 KI group regardless of background. The effect size is calculated as the difference in means divided by the standard deviation of the WT group (Effect Size = (*μ*_MUT_ − *μ*_WT_)/*σ*_WT_); it is measured in units of standard deviation. The heatmap is organized from the anterior of the brain at the top to the posterior at the bottom. Any region in blue is smaller in the mutant versus wild-type and any in red are larger. The asterisk indicates significance at a *q* < 0.05, The dot indicates a trend at *p* < 0.05. *Slc6a4* KO (*N* = 39, 19 WT (9M, 10F), 20 KO (10M, 10F); *Slc6a4* Ala56 KI (B6) (*N* = 40, 20 WT (10M, 10F), 20 KI (10M, 10F); *Slc6a4* Ala 56 KI (129) (*N* = 42, 21 WT (11M, 10F), 21 KI (11M, 10F)

Of the 159 regions examined, no significant differences were found in the *Slc6a4* Ala56 KI mice (Fig. 2). This was seen in the full group or individually in the males and females and was measured with either absolute or relative volume and compared to their corresponding WT. There were, however, some regions within the cerebellum, specifically in the gray matter of the cerebellar vermis, that had trends towards an increase compared to WT with *p* values less than 0.05, but these results did not survive the correction for multiple comparisons (Fig. 2). In the *Slc6a4* KO mice, we see 47 out of the 159 regions have absolute volume differences compared to WT in the full group; again, this is driven primarily by the females, which have 69 regional differences while the males have 0. The smaller regions in the female mice are located in the cortex, specifically in the piriform, auditory, temporal association, and entorhinal cortices, as well as large scale decreases throughout the cerebellum (Fig. 2). Despite the lack of findings for the female *Slc6a4* KO mice with relative volume in the summary regions, there were specific areas where differences were found, including the auditory, ectorhinal, and entorhinal cortices as well as the cortical amygdaloid area (Fig. 2). Furthermore, increases in relative volume were found in the cerebral peduncle and lateral septum.

**Fig. 3:**
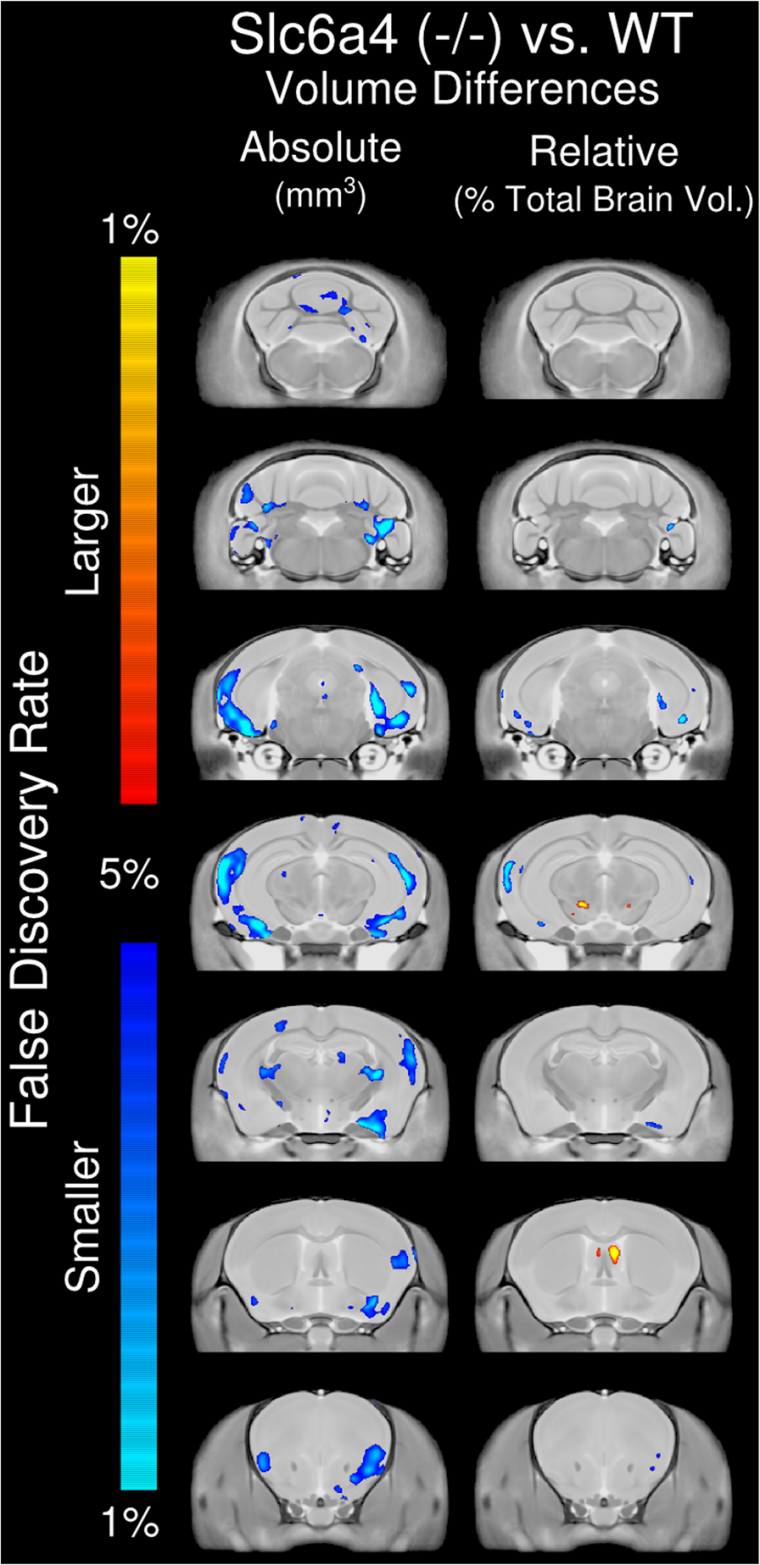
This figure shows the significant voxelwise differences between the *Slc6a4* KO and its corresponding WT. Anything shown in blue/cyan is significantly smaller than the WT and anything in red/orange is significantly larger. *Slc6a4* KO (*N* = 39, 19 WT (9M, 10F), 20 KO (10M, 10F)

Voxelwise analysis also found no significant differences between the two *Slc6a4* Ala56 KI mice and their WT counterparts on either background. This was true for both absolute and relative volume measures. Similar to the regional findings, the full group of *Slc6a4* KO mice on the B6 background highlighted several areas of significance. Figure 3 shows the significant voxelwise differences in both absolute and relative volume in the *Slc6a4* KO. Absolute volume decreases were seen in the lateral cortex, amygdala, and areas of the thalamus, and relative volume increases were seen in the lateral septum and thalamus.

**Fig. 4:**
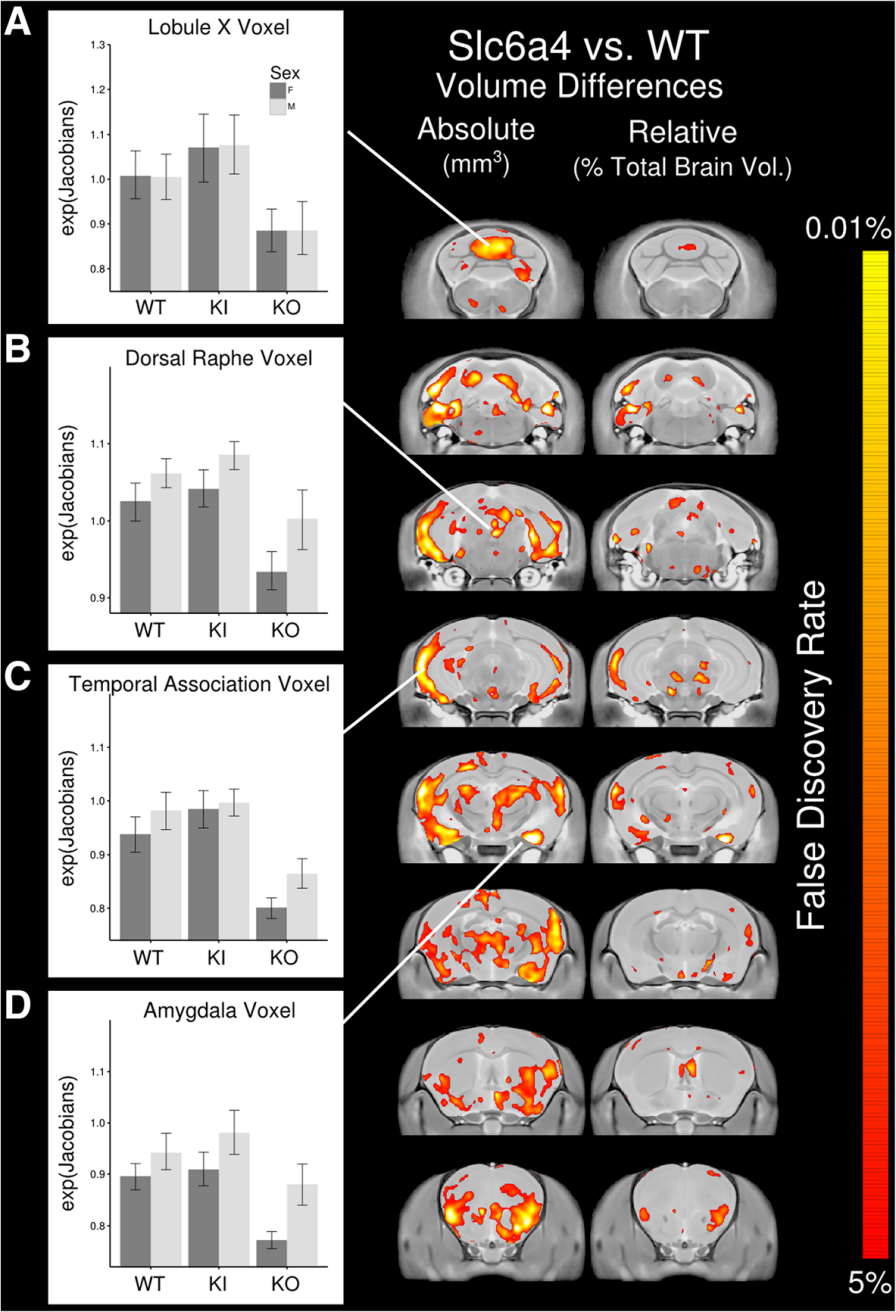
**a**–**d** This figure shows significant voxelwise differences as measured using an ANOVA while factoring in both the sex and background of the mice. All 121 mice were used in this comparison. The panel on the right displays a coronal flythrough highlighting the location of volume differences found due to the *Slc6a4* gene in both absolute and relative volume. For this figure, the C57Bl6/J mice from the Slc6a4 KO group were normalized to the C57Bl6/J mice from the Slc6a4 Ala56 KI group (see the “Methods” section). *Slc6a4* KO (*N* = 39, 19 WT (9M, 10F), 20 KO (10M, 10F); *Slc6a4* Ala56 KI (B6) (*N* = 40, 20 WT (10M, 10F), 20 KI (10M, 10F); *Slc6a4* Ala 56 KI (129) (*N* = 42, 21 WT (11M, 10F), 21 KI (11M, 10F)

Further assessment of the variance across all groups, after normalization of the *Slc6a4* KO group (see the “Methods” section), revealed several areas affected both voxelwise and regionally. Figure 4 shows the significant differences due to the *Slc6a4* gene assessed by an ANOVA while factoring in both the background strain and sex. Four different example voxels are shown on the left (Fig. 4a–d): lobule X, the dorsal raphe, temporal association cortex, and the amygdala. The voxelwise differences recapitulate the differences seen in Fig. 3 but also add a number of new areas of interest, particularly in the colliculi and more widespread differences in the orbital frontal cortex, thalamus, and hypothalamus (see Additional file 1: Table S1 for a full listing of the absolute and relative volume differences seen in Fig. 4). A specific region of interest, relevant to the serotonin system, is the dorsal raphe nuclei, not included in our regional atlas but clearly highlighted.

**Fig. 5:**
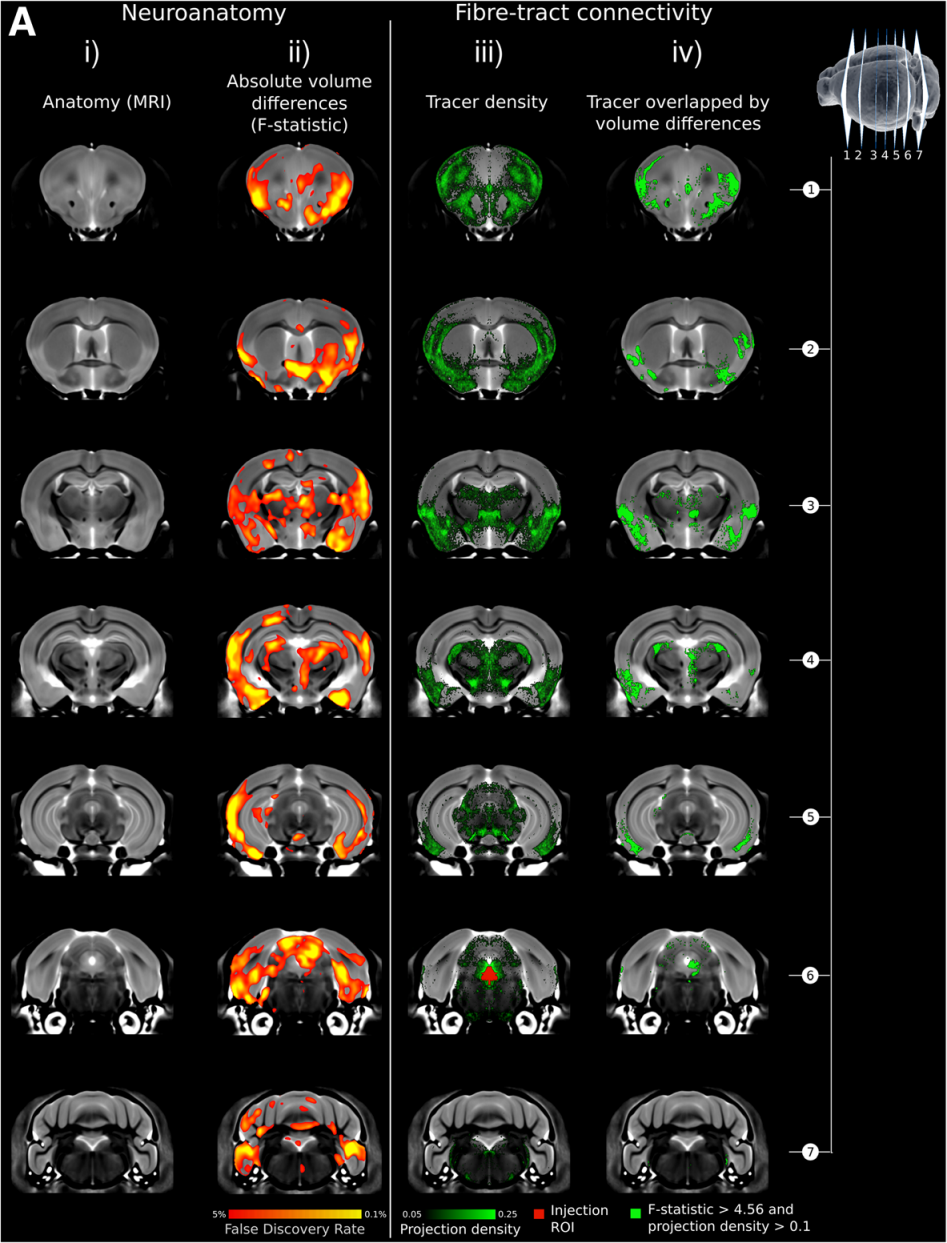
Association between the dorsal raphe nucleus (DRN) fiber-tract connectivity and SERT volume differences. **a** Shown in vertical panels are coronal slices through the mouse brain from anterior (top row) to posterior (bottom row), see top right of figure for location of slices relative to brain. Vertical panel columns correspond to **i)** anatomy (via MRI), **ii)** absolute volume differences between groups (*F*-statistic), **iii)** neuronal tracers projecting anterograde from the dorsal raphe nucleus, and **iv)** voxels which show significant differences at a FDR *q* < 0.05 (*F* > 4.56) that also express a tracer signal

Figure 5 shows comparisons between the volume differences assessed in Fig. 4 and the neuronal projection data for the DRN from the Allen Institute. A comparison of the *F*-statistic map showing *Slc6a4* volume differences and projections from the DRN (Fig. 5a, columns ii and iii) shows that anterograde projections overlap significant *F*-statistic voxels for some frontal cortex areas; however, for most other regions, projections come near to, but do not overlap with regions that show significant differences in volumes (Fig. 5 column iv). Specifically, the amygdala, anterior hypothalamus, and auditory areas of the temporal cortex, along with the superior colliculus, contain significant volumetric differences and have projections from the DRN nearby but do not overlap.

## Discussion

Gain of function SERT manipulations is thought to have a minimal effect on the anatomy of the brain [56]. It has, however, been shown previously that mice with behavioral phenotypes also feature neuroanatomical phenotypes in 87% of cases [57]. Therefore, there is often an expectation that known behavioral findings will be matched by a mesoscopic anatomical difference. The Slc6a4 Ala56 KI mice have differences in ultrasonic vocalizations (increased for B6 background, decreased for 129) and social interaction, as assessed by the three chamber tests (129S6) [36, 37]. The *Slc6a4* KO mice have increased anxiety, increased stress, decreased aggression, increased acoustic startle, decreased exploratory behavior, and decreased motor agility [32, 58]. Due to the known behavioral phenotypes, robust neuroanatomical findings were expected across the models. Additionally, as MRI findings tend to be very sensitive, with several studies showing replications across labs (BTBR [59, 60], Neuroligin3 R451C knockin [61, 62]), and differentially created models (16p11.2 CNV [63, 64]), the relatively modest neuroanatomical findings due to modifications in the SERT gene were unexpected.

A recent examination of 26 different mouse models of autism, which included gene deletions, modifications, and duplications, noted that 70%, or 18 of the 26 models examined, were found to have either a significant relative or absolute volume difference [40]. While it should be noted that the data from the male mice used in this study were the same as the *Slc6a4* KO and *Slc6a4* Ala56 KI mice used in that study, none of the female mice used here were included. With 800+ genes associated with autism [16, 17], there are bound to be some null results or undetectable differences at the mesoscopic scale used in this work. The MRI analysis performed here is not at cellular resolution; therefore, any differences at a microscopic scale or the cellular level would be undetectable at the 56-μm isotropic voxel size used. This includes the known deficits in the organization and differentiation of the *Slc6a4* KO somatosensory whisker barrel cortex [65]; no differences were found in the somatosensory cortex in any sex, measure, or model, which highlights a known functional/organizational difference that is undetectable by volume at the mesoscopic scale of the MRI.

The *Slc6a4* Ala56 KI mutation by itself has no effect on the mesoscopic neuroanatomy, only trends, since no significant differences were found on either background in either absolute or relative volume when compared to its own wild-type. The *Slc6a4* Ala56 KI mutation is a gain of function mutation that causes hyperserotonemia, more so on the 129 backgrounds than B6 [36]. It was also noted that the 5-HT levels in the *Slc6a4* Ala56 KI (129) midbrain and forebrain were unchanged [36], which was not true in the *Slc6a4* KO where there was a reduction in intracellular tissue 5-HT level throughout the brain and in serotonergic cell number in the dorsal raphe nucleus [31]. Therefore, it is likely that the Ala56 KI alone does not produce an *Slc6a4* difference large enough to modify neuroanatomy to a detectable degree with the current methodology. As 5-HT is necessary for early brain development [29], there is also the possibility that at postnatal day 60 we are missing early brain developmental differences. Therefore, a longitudinal investigation would be beneficial to track brain development to see if the developmental trajectories are modified by the changes in *Slc6a4* function. Importantly, it was recently shown that maternal Ala56 genotype impacts forebrain 5-HT levels and thalamocortical projections during midgestation [66], with no observed differences driven by embryo genotype. The *Slc6a4* Ala56 KI animals studied here derive from heterozygous dams and sires so that WT and *Slc6a4* Ala56 KI animals could be generated from mothers of the same genotype. This suggests that future studies should examine the impact of the maternal 5-HT system, which was held constant in this study by the use of heterozygous crosses to generate homozygous *Slc6a4* Ala56 KI mice and wild-type littermate controls.

Regardless of the lack of findings individually for the *Slc6a4* Ala56 KI, Fig. 4 clearly shows there is a significant effect of modifying the *Slc6a4* gene on neuroanatomy across all models used here. The *Slc6a4* Ala56 KI model, which is a gain of function mutation, has the opposite effect of the *Slc6a4* KO mutation in several areas throughout the brain (Fig. 4). For example, the dorsal raphe nucleus, the heart of serotonin function in the brain, trends towards an increase in size for the *Slc6a4* Ala56 KI and decreased for the *Slc6a4* KO (Fig. 4b) but is significant across the entire study. Therefore, we would speculate that a gain or loss in function of SERT corresponds to a gain or loss in volume in several regions in brain, likely originating with the dorsal raphe. In rats and non-human primates, it has been shown that the efferent projections from the rostral group of the raphe nuclei, which includes the dorsal raphe nucleus, ascend through the internal capsule to the lateral cortex and additionally travel through the medial forebrain bundle to the hypothalamus, basal forebrain, and amygdala [1, 5–8]. The internal capsule is also significantly affected by genotype (*F* = 6.55, *q* < 0.01), as well as a large area in the lateral cortex (Fig. 4). Similarly, the hypothalamus (*F* = 4.05, *q* = 0.04), basal forebrain (*F* = 6.37, *q* = 0.01), and amygdala (*F* = 9.54, *q* = 0.001) are also significantly affected by genotype. This indicates that the changes to the *Slc6a4* gene have an impact on the targets of projections from the rostral group of the raphe nuclei. The differences seen here in opposing directions for the *Slc6a4* Ala56 KI versus the *Slc6a4* KO in several regions (Fig. 4), additionally, are consistent with recently published work showing that these genotypes have opposing functional and developmental effects in the gut [67].

These findings were examined further by comparing our volumetric findings with neuronal projection data from the DRN. It was expected that there would be an overlap between the volume differences seen (Figs. 4 and 5ii) with the projection density images of the neuronal tracer seen in Fig. 5iii. While some areas did show overlap, particularly in the frontal cortical regions, for most areas, the tracer appears to come near to the volumetric differences but does not overlap directly, potentially reflecting the role of 5-HT as a morphogen acting via broader diffusion or volume transmission during development [68, 69]. The DRN projections do, however, project towards several of the affected regions albeit not within them, as visualized by the Allen Brain Atlas data. It is possible, therefore, that the differences in SERT in the DRN in both the *Slc6a4* KO and *Slc6a4* Ala56 KI are in fact causing these volumetric differences in the adjacent regions through this projection.

Of additional interest was the finding of a stronger neuroanatomical phenotype in the female *Slc6a4* KO mice, and to a lesser insignificant extent in the *Slc6a4* Ala56 KI mice, compared to the males. The stronger phenotype in the females is intriguing, and it seems to match up well with a study that reported an increase in 5-HT levels in female rats compared to their male counterparts [70]. This higher level of 5-HT in the females could possibly make them more susceptible to the knockout or knockin of SERT function. This highlights the necessity for investigation of both male and female mice in these types of studies and also lends additional weight to the recent NIH insistence of inclusion of both sexes in biomedical research [71]. Our volumetric findings in females contrast with a previous report that heterozygous male and female *Slc6a4* KO mice (+/−) have increased brain mass, normalized to body mass, compared to controls at 8–12 weeks [72]. It is difficult to compare such different methodological approaches, since the disparity could be driven by genotype differences (+/− versus −/−), differences in age, differences driven by body mass, weight versus volumetric analyses, or removal of the brain from the skull versus in skull neuroimaging.

Future work could benefit from the examination of neuroanatomical differences at earlier timepoints, whether done longitudinally or as a cross-sectional study. Further, examination of the effects of maternal genotype, or maternal-offspring genotype interactions, could reveal broader effects on neurodevelopment due to in utero effects. It would also be worth examining additional genetic modifications of the *Slc6a4* gene to see if they follow a similar pattern to what is shown here with increasing brain volume with increase 5-HT uptake. The *Slc6a4* Ala56 KI variant was found to have a ~ 30% increase in 5-HT uptake, but there are additional variants that have up to ~ 70% increases [28, 73]. Therefore, it may be beneficial to see if further increases in the areas mentioned here could be found in these other variants. Similarly, one could also examine the heterozygous *Slc6a4* KO, which may show an intermediate loss of volume compared to WT.

There were a few limitations to this study: (1) It has been shown previously in the literature that there are differential effects of the SERT manipulation on the behavior in these different models. Therefore, this implies that there is likely no correlation between the behavior deficits and the neuroanatomical differences seen here. However, since behavior was not tested in these individual mice, this was beyond the scope of this current work. (2) The control animals used for the *Slc6a4* KO mice were not the ideal controls for this work. The *Slc6a4* KO mice were purchased directly from Jackson Laboratory (JAX #008355), and at that time they were bred either homozygote × homozygote or heterozygous × homozygous, both of which do not allow wild-type littermates. Therefore, for controls, C57Bl6/J mice were used as suggested from Jackson Labs. Ideally, one would want to breed heterozygote × heterozygote in order to get WT littermates for comparison. However, the differences, only seen in females, do seem to indicate that C57BL/6J is a reasonable control neuroanatomically.

## Conclusions

As suggested previously, this work shows that the SERT activity does contribute to adult neuroanatomy. An increase or decrease in the *Slc6a4* function can cause a differential effect on the neuroanatomy in 5-HT relevant regions, particularly in the raphe nuclei. Considering the small number of 5-HT neurons found within the brain and the localization of those neurons, it is clear that serotonin function can be an integral part of the neuroanatomical organization.

## Additional file

Additional file 1: Regional volume differences found for the whole group ANOVA analysis shown in Fig. 4. (XLSX 27 kb)

## Abbreviations

5-HT: Serotonin
5-HTTLPR: Serotonin transporter functional length polymorphic repeat
Ala56: Knockin of an alanine at codon 56
ANTs: Advanced normalization tools
ASD: Autism spectrum disorder
CNV: Copy number variant
DRN: Dorsal raphe nucleus
FDR: False discovery rate
FOV: Field of view
FSE: Fast spin echo
ItgB3: Integrin beta 3
KI: Knockin
KO: Knockout
MRI: Magnetic resonance imaging
OCD: Obsessive compulsive disorder
PBS: Phosphate buffer solution
PFA: Paraformaldehyde
SERT: Serotonin transporter
Slc6a4: Solute carrier family 6 member 4, serotonin transporter gene
T2: Transverse relaxation time
TE: Echo time
TR: Repetition time
VNTR: Variable number of tandem repeats
WT: Wild-type
